# Utility and safety of epilepsy monitoring unit in an inpatient psychiatric setting in Japan

**DOI:** 10.1016/j.ebr.2025.100744

**Published:** 2025-01-29

**Authors:** Go Taniguchi, Mao Fujioka, Yumiko Okamura, Minako Miyagi, Kenichi Yano, Shinsuke Kondo, Kiyoto Kasai

**Affiliations:** a Department of Neuropsychiatry, The University of Tokyo Hospital, Japan; b Nursing Department, The University of Tokyo Hospital, Japan; c Department of Rehabilitation, The University of Tokyo Hospital, Japan

**Keywords:** Epilepsy, Psychogenic non-epileptic seizure, Epilepsy monitoring unit, Long-term video electroencephalography psychiatry

## Abstract

An epilepsy monitoring unit (EMU) with long-term video electroencephalographic monitoring (LTVEM) was launched in our psychiatric ward to facilitate close examination of patients with epilepsy and offer hospital experience to psychiatrists unfamiliar with epilepsy care in Japan. This study aimed to examine the usefulness and safety of the EMU in an inpatient psychiatric setting in Japan by retrospectively reviewing the clinical notes of EMU admission cases in the psychiatric ward of the University of Tokyo Hospital between August 2014 and March 2020. The usefulness of the EMU was evaluated in terms of 1) habitual seizure recordings, 2) change in diagnosis, 3) frequency of epileptic seizures, and 4) surgical cases after EMU admission, and 5) EMU safety. Habitual events were recorded during LTVEM in 69 % of patients. The diagnosis was changed post-EMU evaluation in 28 % of the patients admitted for differential diagnosis. Among 41 patients who received anti-seizure medications, seizure frequency improved in 22 (53 %), whereas 10 (24 %) became seizure-free, and seizures were reduced by >50 % in 12 (29 %). Eleven patients eventually underwent surgery for epilepsy. Among the total 134 patients, 13 (10 %) experienced adverse events during EMU stay and 6 experienced falls, but none required prolonged hospitalization. Furthermore, three patients had panic attacks, but no post-ictal psychiatric symptoms were observed. Admission to an EMU in the psychiatric setting can facilitate accurate diagnosis and improve epilepsy management. Fall prevention measures should be implemented during and after LTVEM for safer EMU evaluations.

## Highlights

• An EMU with LTVEM was launched in Japanese psychiatric ward.
• Habitual events were recorded during LTVEM in 69 % of patients.
• An EMU in the psychiatry setting can facilitate accurate diagnosis.
• An EMU in the psychiatric setting can improve epilepsy management.
• Fall prevention measures should be performed during and after LTVEM for safer EMU.

## Graphical abstract

**Figure:**
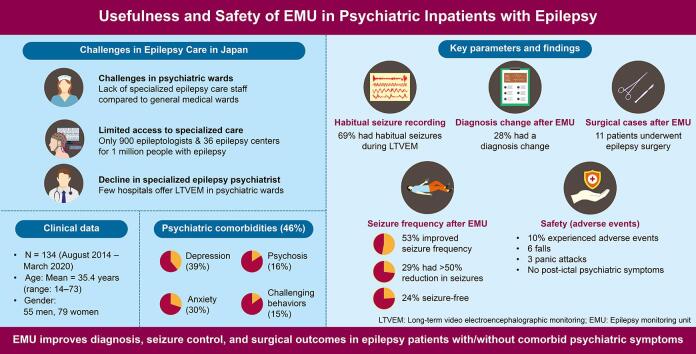

## 1 Introduction

People with epilepsy (PWE) exhibit a higher rate of comorbid psychiatric symptoms than the general population [1]. Comorbid psychiatric symptoms negatively affect the quality of life of PWE, and appropriate treatment is difficult owing to the widely varying etiology of psychiatric symptoms. PWE may experience psychiatric symptoms preceding the seizure (pre-ictal), following the seizure (post-ictal), independently of seizure occurrence (interictal), or as an expression of the seizure (ictal) [2]. Moreover, anti-seizure medications (ASMs) can adversely impact mood, behavior, and cognition [3]. Therefore, for proper treatment of psychiatric symptoms in PWE, psychiatrists must have access to detailed and accurate information about epileptic seizures (ES) and ASM.

However, Japan has only a few epileptologists and epilepsy centers. Compared with 256 epilepsy centers for an estimated 3.4 million PWE in the United States [4], Japan has 36 epilepsy centers for 1 million PWE [5]. Consequently, not all PWE are correctly diagnosed and treated in Japan.

**Fig. 1:**
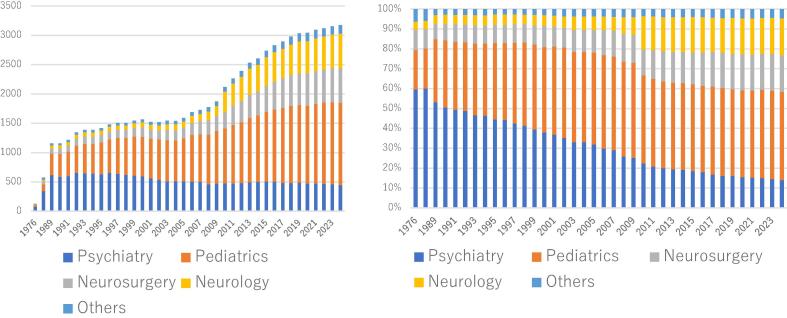
Number of the members in the Japan Epilepsy Society (JES) and percentages of these members by department. The figure on the left shows the number of the members of the JES since 1976; as of September 2024, there are 3,176 members. The figure on the right shows the percentage of the JES member by department: psychiatrists represent 60% of the membership at the time when the JES is founded in 1976, but this percentage has decreased to 14 % as of 2024. (Based on materials submitted at the General Meeting of the Board of Trustees of the JES held in September 2024).

Moreover, epilepsy care in Japan has a unique history and practice structure. Currently, adults with epilepsy are treated in three departments—neurology, neurosurgery, and psychiatry. Psychiatrists have historically played a central role in diagnosing and treating seizures and psychiatric symptoms in adults with epilepsy (Fig. 1) [6]. However, the number of psychiatrists specializing in epilepsy is declining, and only a few hospitals routinely perform long-term video electroencephalographic monitoring (LTVEM) in psychiatric wards.

Therefore, we launched an epilepsy monitoring unit (EMU) with LTVEM in the department of psychiatry at our university hospital with expectations that EMU admission in a psychiatric ward would 1) facilitate access to close examination of PWE with/without psychiatric symptoms and PWE with epilepsy who require differentiation from psychiatric symptoms, 2) provide hospital experience in epilepsy management to young psychiatrists, and 3) offer comprehensive support to all PWE through the existing multidisciplinary teams of psychologists, occupational therapists, and social workers in psychiatric wards.

EMUs must be designed to be efficient and effective and guarantee patient safety and well-being [7]. However, whether EMUs in psychiatric wards are as effective and safe as those in general wards remains unclear. In our hospital, although two (G. T. and M. F.) EMU key members were psychiatrists and certified epileptologists, the other members were inexperienced in epilepsy care. Unlike in Europe and the United States, many hospitals in Japan do not have dedicated nursing staff and video-electroencephalogram (EEG) technicians. Furthermore, psychiatric wards in Japan operate with fewer nurses than general wards. Despite these limitations, comparable efficacy and safety of EMUs in psychiatry to those of EMUs in general wards may be beneficial for both patients and young psychiatrists, who could gain effective training through EMUs. Therefore, this study aimed to examine the usefulness and safety of EMUs in an inpatient psychiatric setting in Japan.

## 2 Methods

### 2.1 Setting

This study was conducted in the EMU of the psychiatric ward at the University of Tokyo Hospital, a training hospital accredited by the Japan Epilepsy Society. Originally, this hospital had an EMU with two beds in its neurosurgery ward and one EMU with one bed in the pediatric ward, with an EMU established in the psychiatry ward in 2014. For adult patients, EMU was performed for pre-surgical evaluation purposes in the neurosurgical ward and differential diagnosis or seizure classification purposes in the psychiatric ward. Notably, due to the absence of epileptologists in the neurology department, no EMU was established in the neurology ward at that time. The psychiatric ward consisted of a 21-bed open ward and a 27-bed closed ward, each with 30 beds prior to the ward’s relocation in January 2018. One room in the open ward was allocated to the EMU before and after the ward relocation.

The psychiatric EMU was managed by a team of one or two epileptologists and two or three general psychiatrists. Epileptologists developed a plan for EMU admission and were responsible for all decisions regarding patients in EMUs during the hospitalization period. Patients underwent psychiatric and neurological examinations and LTVEM analysis. If any staff members in the psychiatric ward had concerns about the patient’s safety and care, general psychiatrists were on first call day and night to examine the patient. Non-EMU dedicated nurses provided bedside care and performed an ictal examination on the patient with a patient-to-nurse ratio of 3:1 during the day and 10:1 during the night. Although no EEG technicians were available for 24 h, EEG technicians in the hospital’s laboratory department were also responsible for the EMU technical aspects and were on call during the daytime. Paramedics, including psychologists, social workers, and occupational therapists, interviewed patients and/or family during and after LTVEM to comprehensively evaluate patients admitted to the EMU.

### 2.2 Participants

The study cohort consisted of consecutive patients admitted to the EMU of the psychiatric ward of the University of Tokyo Hospital between August 2014 and March 2020. None of the patients had multiple admissions to the EMU during this period. Patients were referred from various departments, both inside and outside the hospital, and were examined by an epileptologist (G. T.) before admission to determine LTVEM need. The primary purpose of EMU admission was to evaluate seizure symptoms and not to diagnose or treat comorbid psychiatric symptoms. The purpose of the EMU was to allow 1) differential diagnosis to determine whether paroxysmal spells were ES (differential diagnosis group), 2) seizure/syndrome classification to select an optimal treatment for patients with a confirmed diagnosis of epilepsy (seizure classification group), and 3) pre-surgical evaluation of candidates for epilepsy surgery who were difficult to examine in the neurosurgical ward (pre-surgical evaluation group).

### 2.3 Procedures

#### 2.3.1 Patient companion and EMU environment

Written informed consent was obtained from all patients after explaining the aim of the procedure and its potential risks. Family members were encouraged to stay overnight during LTVEM. Just before LTVEM began, nurses educated companions according to a standardized protocol on how to: (1) press the nurse’s call button quickly after noticing symptoms indicative of seizure, (2) remove patients' blanket to allow better visualization of extremity movements during a seizure, (3) not obscure patient view during the seizure, and (4) assist the patient when at risk of falling. In the few cases where family members could not accompany the patient, EMU staff considered implementing individual safety measures, including the non-reduction of ASMs to avoid seizures leading to injury or sequelae. No physical restraints were used to prevent falls. All patients were required to stay in a bed with thick padded side rails in the upright position while undergoing LTVEM. The bed was locked and secured at a low position. Graduated compression stockings were used in patients at risk of developing deep venous thrombosis. Patients with suspected hyperkinetic seizures could sleep on a floor mattress.

#### 2.3.2 EMU design and equipment

The EMU was equipped with a 32-channel digital VEEG system (Neurofax EEG-1200, Nihon Kohden Co., Tokyo, Japan). Surface EEG electrodes were placed according to the international 10–20 system with anterior temporal (T1 and T2) electrodes. The EMU also had remotely adjustable color video cameras with infrared imaging for nocturnal monitoring under low-light conditions. Continuous EEG and video were streamed to the monitoring screen at the nurse station, and the general psychiatrists or nurses intermittently monitored the screens. The members of staff immediately went to the EMU room to examine the patient when observed seizure symptoms or safety issues occurred. Nurses provided bedside care and neurological function testing during and after the ictal state following a standardized seizure protocol established within the hospital. Cardiac monitoring and pulse oximetry were available as further safety measures.

To identify seizures, ASMs were tapered or discontinued as needed, and some patients underwent sleep deprivation and photic stimulation at epileptologist’s discretion. The rate of ASM withdrawal ranged from discontinuation on the day of admission to a reduction of half the dose over 5 days. No standard protocol was available for ASM withdrawal. ASM withdrawal was personalized considering both drug and patient-specific factors, such as drug efficacy, medication half-life, seizure frequency, history of status epilepticus (SE), and risk of withdrawal seizures. The epileptologist reviewed video-EEG recordings daily for missed and subclinical seizures. The recorded seizures were analyzed by a general psychiatrist under the epileptologist’s supervision. In some cases, LTVEM was completed earlier than scheduled at the epileptologist’s discretion if enough target seizures were recorded or if the patient requested it.

#### 2.3.3 Seizure response

In case of unexpected seizure clusters or SE due to ASM reduction, the standard protocol involved intravenous injection of diazepam as first-line treatment and intravenous drip of fosphenytoin as second-line treatment. Seizure clusters were defined as three or more focal seizures with impaired awareness or two tonic–clonic seizures occurring within 4 h [8], [9], [10]. SE was defined as convulsive seizures lasting longer than 5 min, or when consciousness was not regained between two consecutive seizures [10].

In the event of a seizure cluster, staff carefully evaluated psychiatric symptoms to facilitate the prompt transfer of the patient to a less stimulating room in the psychiatric ward in the post-ictal psychosis cases. If a patient experienced a prolonged seizure or seizure cluster, and the nurse suspected psychogenic non-ESs (PNES) or ES, a general psychiatrist examined the patient at the bedside, in the presence of or under the remote supervision of an epileptologist.

Although the seizure protocol for prolonged PNES was shared with the patient and companions in advance, the epileptologist explained that the events were non-epileptic and would cause no serious sequelae. ASM was not administered, and oxygen was provided as needed when prolonged PNES occurred.

#### 2.3.4 Additional tests after LTVEM for comprehensive evaluation

Following LTVEM, unaccompanied patients remained hospitalized and underwent imaging and psychological examinations for comprehensive seizure evaluation. Psychological tests and interviews were not performed when the patient was feeling unwell due to seizures or test fatigue. Comprehensive tests performed during hospitalization are less economically burdensome for patients and their families, as Japan’s public medical insurance system allows patients to reduce their monthly fees to a certain limit by applying in advance. If ASMs were tapered or discontinued during LTVEM, patients were restarted on their outpatient ASM doses if these were to be continued on discharge. ECG and pulse oximeter monitoring were mandatory during this period, and video monitoring in the patient room was continued as needed.

If habitual seizures were not recorded during LTVEM or recorded seizures were difficult to interpret, a comprehensive diagnosis was made based on EEG findings during the interictal period, imaging studies, psychiatric/psychological evaluation, and medical history, including post-LTVEM follow-up.

#### 2.3.5 Explanation at discharge

The results of the tests performed during hospitalization were explained to the patients and their families on the day of discharge. An ictal video-EEG was used for explanation, unless the patient refused. The social worker and occupational therapist were present with the psychologist to discuss the support system after discharge, if psychosocial support was needed. Whether the habitual seizure was an ES or a PNES, patients or caregivers were asked to maintain a seizure diary. Seizure frequency was assessed using the seizure diary or the results of the interviews at every visit.

### 2.4 Study design

This study retrospectively reviewed the medical charts of the patients admitted to the EMU. Psychiatric symptoms were diagnosed by psychiatrists using a non-structured interview during EMU admission. The presence of intellectual disability (ID) was determined based on the results of psychological examinations (the Wechsler Adult Intelligence Scale or Wechsler Intelligence Scale for Children) conducted during EMU admission or officially issued ID certificates. Patients with borderline intelligence (IQ 71–84) were classified as the “ID group,” as they often required dedicated support for their social activities. Physical disability was assessed using officially issued disability certificates.

To evaluate EMU usefulness, this study 1) investigated the seizures recorded during LTVEM and the relationship between seizures and demographic and clinical data, 2) examined the number of patients in the differential diagnosis group whose diagnoses were confirmed or changed, 3) compared seizure frequencies in the seizure classification group at 6 months before and after EMU stays, and 4) examined the number of surgical procedures.

To confirm safety, the presence and nature of adverse events (AEs) in all cases were reviewed. Statistical tests were conducted to determine an association between AEs and each indicator. The following were considered as AEs: seizure clusters or SE, falls, orthopedic injury, serious cardiac abnormalities (such as asystole, severe bradycardia, or tachycardia), thrombotic events, respiratory complications, and psychiatric symptoms (such as post-ictal psychosis, panic attacks, or post-ictal aggression).

### 2.5 Statistical analysis

For categorical variables, chi-square or Fisher’s exact tests were performed according to the expected frequency. For univariate analysis, a parametric (Student’s *t*-test) and nonparametric (Mann–Whitney) test were performed for normally and non-normally distributed variables, respectively. P < 0.05 was considered statistically significant. All statistical analyses were performed using IBM SPSS ver. 25 (IBM Corp., Armonk, NY, USA).

### 2.6 Ethical considerations

Written informed consent and opt-outs were obtained for this study. Patients who did not provide consent to participate in the study were excluded. This study adhered to the ethical principles of the Declaration of Helsinki and was approved by the Ethics Committee of the University of Tokyo Hospital (approval date: April 18, 2018; approval number: 11898).

## 3 Results

### 3.1 Demographic and basic clinical data

Among 140 patients admitted to the EMU during the study period, 134 (55 men and 79 women; mean age 35.4 years, standard deviation [SD]: 13.9, range: 14–73 years) who consented for participation were included (Table 1). Psychiatric comorbidities were diagnosed in 62 (46 %) patients as follows: depression (39 %, 24/62), anxiety (31 %, 19/62), psychosis (16 %, 10/62), and challenging behaviors (15 %, 9/62). Eighty patients (60 %) had borderline (31 %, 25/80), mild (45 %, 36/80), moderate (18 %, 14/80), or severe ID (6 %, 5/80). Nine patients (7 %, 9/134) had physical disabilities, including seven with paralysis, one with vision loss, and one with dysphagia.

**Table 1:** Demographic and basic clinical data.

N = 134		
Gender: male/female (%)		55 (41 %)/79 (59 %)
Age on admission: mean (SD)		35.4 (13.9)
Intellectual disability		80 (59 %)
	borderline	25
	mild	36
	moderate	14
	severe	5
Physical disability		9 (7 %)
	paralysis	7
	vision loss	1
	dysphagia	1
Psychiatric comorbidities		62 (46 %)
	depression	24
	anxiety	19
	psychosis	10
	challenging behaviors	9
Referral from	psychiatry	85 (63 %)
	neurology	16 (12 %)
	neurosurgery	18 (13 %)
	pediatric	13 (10 %)
	others	2 (1 %)
Reason for admission	differential diagnosis	85 (63 %)
	seizure classification	45 (34 %)
	presurgical evaluation	4 (3 %)
Seizure frequency	daily	14 (10 %)
	weekly	49 (37 %)
	monthly	58 (43 %)
	yearly	10 (7 %)
	over yearly	3 (2 %)
Numbers of ASM: mean (SD)		2.3 (1.2)
Withdrawal of ASM		115 (86 %)
Length of admission: mean (SD)		11.1 (2.5)
LTVEM only		14 (10 %)
LTVEM + other evaluation		120 (90 %)
Patients for whom support and intervention were provided for psychosocial problems		88 (66 %)
Length of LTVEM: mean (SD)		4.7 (0.9)
Patients with companions during LTVEM		109 (81 %)
Patients followed up by our department		90 (67 %)
Follow up period (month): mean (SD)		40.7 (18.5)

Abbreviations: ASM; antiseizure medication, LTVEM, long-term video electroencephalographic monitoring; SD, standard deviation.

The psychiatry department was the most common referral source for patients (64 %, N = 85), followed by neurosurgery (13 %, N = 18), neurology (12 %, N = 16), and pediatrics (10 %, N = 13). Reasons for EMU admission included differential diagnosis in 63 % (N = 85), seizure classification in 34 % (N = 45), and pre-surgical evaluation in 3 % (N = 4) of patients.

The frequency of seizures at the time of admission was classified as monthly in 58 patients (43 %), followed by weekly in 49 (37 %), daily in 14 (10 %), yearly in 10 (7 %), and less often than yearly in 3 (2 %) patients. Although most patients (N = 120, 90 %) underwent various tests following LTVEM completion, some (N = 14, 10 %) underwent LTVEM only during their EMU hospitalization because of difficulty in staying in the hospital alone due to autism or dementia. Furthermore, 88 patients, 60 with a final diagnosis of PNES and 26 PWE with comorbid psychiatric symptoms, received special treatment and support by a psychiatrist or psychologist. The mean total length of hospital stay was 11 days (SD: 2.5, range: 2–12), and the mean video-EEG duration was 5 days (SD: 0.9, range: 1–7). Notably, 81 % of the patients (N = 109) had a companion during LTVEM, and 67 % of patients (N = 90) were followed up after EMU admission.

### 3.2 Recorded seizures during LTVEM

Ictal events (ES or non-epileptic events) were recorded in 92 patients (69 %), including 55 ES and 41 non-epileptic events (40 PNES and 1 sleep disorder). Four patients had both ES and PNES recorded during LTVEM. Although seizures were recorded in all patients in the surgical study group, no ictal events were recorded in 42 patients (30 in the differential diagnosis group and 12 in the seizure classification group).

The statistical tests revealed that the captured seizure group had significantly more cases of ID (P = 0.007), daily seizures (P = 0.037), and weekly seizures (P = 0.014), whereas the non-recorded group had significantly more yearly seizure events (P = 0.011) (Table 2). In 86 of the 92 patients with a recorded seizure episode, either nurses, psychiatrists, or family members noticed the seizure symptoms and responded per protocol. Despite the presence of clinical symptoms of ES, seizures were missed in six patients, but LTVEM analysis on the same day confirmed them. In contrast, in PNES, every seizure was noticed by some observer and responded to.

**Table 2:** Comparison of the patients with recorded seizures and without recorded seizures during long-term video electroencephalographic monitoring.

	Recorded group (N = 92)	Non-recorded group (N = 42)	P-value
Sex, female	54 (59 %)	25 (60 %)	0.928
Age on admission (years)	36.8	32.2	0.073
psychiatric comorbidities	45 (49 %)	17 (40 %)	0.364
**intellectual disabilities**	**62 (67 %)**	**18 (43 %)**	**0.007**
physical disabilities	9 (10 %)	0 (0 %)	0.057
**daily seizure**	**13 (14 %)**	**1 (2 %)**	**0.037**
**weekly seizure**	**40 (43 %)**	**9 (21 %)**	**0.014**
monthly seizure	37 (40 %)	22 (52 %)	0.188
**yearly seizure**	**3 (3 %)**	**7 (17 %)**	**0.011**
LTVEM duration (days)	4.6	4.9	0.137
companion	78 (85 %)	31 (74 %)	0.130
Withdrawal of ASM	80 (87 %)	35(83 %)	0.577

For categorial variables, we conducted Chi square or Fisher’s exact test according to the expected frequency in the cell. For univariate analysis, we used a parametric test (Student’s *t*-test) when the variables had a normal distribution and a nonparametric test (Mann-Whitney test) for a non-normal distribution. Age on admission and LTVEM duration are shown as means.

Abbreviations: ASM, antiseizure medication, LTVEM, long-term video electroencephalographic monitoring; PNES, psychogenic non epileptic seizure. Significance set at 0.05.

### 3.3 Diagnostic contribution of EMU

**Fig. 2:**
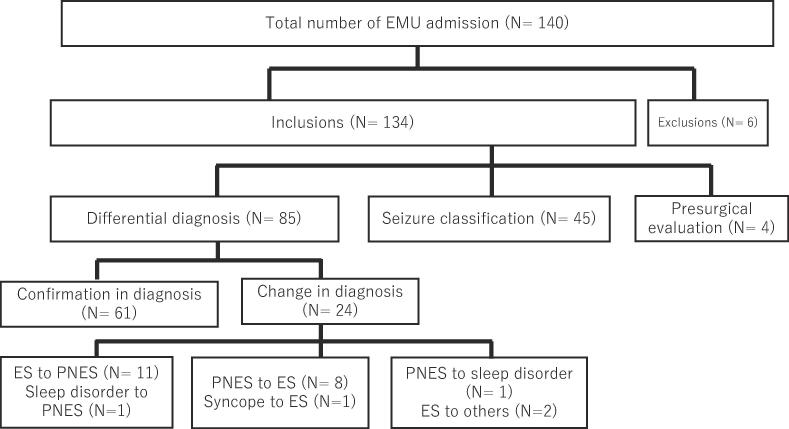
Overview of total number of admissions, reasons for admission, and alternations in diagnosis for patients admitted to the epilepsy monitoring unit in psychiatry in the University of Tokyo Hospital from 2014 to 2020.

Among the 85 patients admitted for differential diagnosis, 24 (28 %) had a change in diagnosis after EMU admission (Fig. 2). No ictal events were recorded via LTVEM in 30 (35 %) of the 85 patients in the differential diagnosis group. Even when seizures were not recorded, the diagnosis was reviewed after the detailed history, EEG findings during the interictal period, psychological testing, and imaging studies were discussed within the EMU team. Consequently, 10 of the 35 patients with no documented events were admitted to the EMU, where their diagnoses were changed.

Eleven patients with a preadmission diagnosis of ES were diagnosed with PNES after EMU evaluation, whereas nine patients with a preadmission diagnosis of non-ESs (eight PNES and one syncope) were eventually diagnosed with ES after EMU admission. Preadmission diagnosis of PNES was changed to sleep disorder in one patient and to other non-epileptic events (non-epileptic behaviors associated with dementia or generalized anxiety disorder) in two patients.

Of 134 patients, 60 (45 %; 27 PNES-only and 33 PNES with epilepsy) were finally diagnosed with PNES (Table 3). Of 60 patients with PNES, 40 were diagnosed via LTVEM (documented PNES), one by an epileptologist directly observing events (clinically established PNES), and one based on information from a nurse witnessing events (probable PNES) following LTVEM. Seventeen patients were diagnosed on information from witnesses, self-reported information, and follow-up observations (possible PNES). A differential diagnosis was made in 85 patients. Fifty-nine patients (70 %; 27 PNES-only and 32 PNES with epilepsy) were eventually diagnosed with PNES (Table 3).

**Table 3:** Diagnostic composition before and after admission to the epilepsy monitoring unit among total patients (N = 134) and patients admitted for differential diagnosis (N = 85).

Diagnosis (N = 134)		before EMU	after EMU	change
	Epilepsy	74	70	−4
	Focal	62	61	−1
	General	4	8	4
	Unclassified	8	1	−7
Nonepileptic events		60	64	4
	PNES-only	26	27	1
	PNES with epilepsy	31	33	2
	Syncope	2	1	−1
	Sleep disorders	1	1	0
	Others	0	2	2

Diagnosis (N = 85)		before EMU	after EMU	change
Epilepsy		26	22	−4
	Focal	20	21	−1
	General	1	1	0
	Unclassified	5	0	−5
Nonepileptic events		60	64	4
	PNES-only	26	27	1
	PNES with epilepsy	30	32	2
	Syncope	2	1	−1
	Sleep disorders	1	1	0
	Others	0	2	2

Abbreviations: EMU, epilepsy monitoring unit; PNES, psychogenic non epileptic seizure.

### 3.4 Impact of EMU result-derived adjusted-ASM on seizure frequency

Among the 45 patients who were admitted for seizure classification, four underwent surgical procedures. The seizure frequency in the 41 patients who continued ASM treatment after EMU evaluation was daily in 4, weekly in 13, monthly in 23, yearly in 4, and less often than yearly in 1. Of 41 patients, 16 had comorbid psychiatric symptoms (39 %), including six, five, four, and one patient with psychosis, depression, challenging behaviors, and anxiety, respectively. After EMU admission, the outcomes for ES included seizure-free in 10 (24 %), more than 50 % seizure reduction in 12 (29 %), no change in 16 (39 %), and exacerbation in 3 (7 %) patients.

### 3.5 Usefulness of EMU as a pre-surgical evaluation

Eleven patients eventually underwent epilepsy surgery, including four for pre-surgical evaluation at the time of admission and seven for other LTVEM purposes (three for differential diagnosis and four for seizure classification). Of 11 patients, four had comorbid psychiatric symptoms (37 %), including two with psychosis, one with depression, and one with challenging behaviors. All the patients underwent surgery without repeated LTVEM. The epilepsy surgeries performed were anterior temporal lobectomy (ATL) in three patients and vagus nerve stimulator (VNS) implantation in eight. Three patients who underwent ATL achieved seizure control after surgery. Three of the eight patients who underwent VNS implantation exhibited seizure improvement, and the condition remained unchanged in five.

### 3.6 AES during EMU admission

#### 3.6.1 Falls

Among 134 patients, 13 (9.7 %) experienced AEs during EMU stays (Table 4). The most common AE was falls, which occurred in six patients, who experienced three falls each during LTVEM and post-LTVEM ambulation. All the falls were minor, and patients did not require any special treatment or prolonged hospital stays. One patient with PNES required further testing with a head CT scan to rule out intracranial bleeding or facial fractures. Five patients who experienced falls had PNES, and one had epilepsy. Of the five patients with PNES who experienced falls, one fell from the bed during LTVEM, and the remaining four fell from a standing position (three while ambulating after LTVEM completion and one while walking under LTVEM recording because the habitual seizure was gait-induced). Companions accompanied five of the six patients who fell. One of the patients with PNES promptly fell from the bed and suffered injuries requiring head CT despite being attended by a family member. Furthermore, one patient with epilepsy had ES and fell during the transfer from the toilet to the bed during LTVEM but did not suffer from serious injuries owing to the support of her family.

**Table 4:** Adverse events and incidents in the epilepsy monitoring unit in psychiatry.

Adverse events		13 (9.7 %)
Fall		6 (4.4 %)
	During LTVEM	3
	Ambulation in the post-LTVEM period	3
Seizure cluster		4 (3.0 %)
Panic attack		3 (2.2 %)

Incidents		4 (3.0 %)

Companion’s upsetting due to prolonged PNES		3
Termination of LTVEM earlier than scheduled		1

Abbreviations: LTVEM, long-term video electroencephalographic monitoring; PNES, psychogenic non epileptic seizure.

#### 3.6.2 Seizure clusters and SE

Only two motor seizure and two non-motor seizure clusters were observed, both of which were controlled by first- or second-line management. No SE was observed in the cohort.

#### 3.6.3 Psychiatric and other events

Three patients experienced panic attacks at the end of the study period. Two of the three patients had comorbid anxiety disorders and were taking psychotropic medication before EMU admission. The panic attacks were relieved without pharmacological intervention, and LTVEM was performed as scheduled. The patients rarely experienced panic attacks in their daily lives and did not experience them during outpatient follow-up after discharge. No cardiac abnormalities, thrombotic events, or respiratory complications were observed.

#### 3.6.4 Statistical analysis of AEs

The statistical analysis revealed difference in the associations between AE and psychiatric comorbidities, length of LTVEM, presence of companion, and ASM withdrawal between the AE and non-AE groups (P = 0.993, 0.764, 0.461, and 1.000, respectively) (Supplemental File 1).

#### 3.6.5 Incidents

Four incidents occurred during LTVEM, although they did not result in AEs. LTVEM was terminated early in one case because the patient could not tolerate it and in three cases because of upset companion family members following a prolonged PNES. If PNES was suspected, the pre-determined protocol to not treat with ASMs, which was shared with the patient and their family prior to the LTVEM, was implemented. In cases of prolonged PNES without motor symptoms, family members tended to be less psychologically upset in the EMU. However, prolonged PNES with motor symptoms made them psychologically upset, even though LTVEM could be performed as scheduled.

## 4 Discussion

This study evaluated the usefulness and safety of using an EMU in an inpatient psychiatric setting in Japan. Habitual ictal events were recorded in 69 % of patients. Of the patients admitted for differential diagnosis, diagnosis was changed in 28 % after EMU admission. EMU provided useful information for appropriate treatment, with 53 % of the ASM treatment group and 55 % of the surgical group showing improvement in seizure control. AEs were observed in 9.7 % of patients, with the most common being falls in six patients (4.4 %) that did not lead to serious consequences. Four seizure clusters occurred, but all were controlled by protocolled treatments. This study demonstrates that EMU admission could facilitate accurate diagnosis and management of epilepsy in a psychiatric as well as a general setting. Meanwhile, the study also indicates the need to strengthen fall prevention measures to guarantee safety.

Sixty percent of patients admitted to the psychiatric EMU had comorbid ID. A previous study reported ID in 23 % of 398 patients who visited an outpatient epilepsy clinic in Japan [11], a rate lower than that in the present study. This may be attributed to the large number of patients with comorbid psychiatric symptoms (ID is a risk factor for psychiatric disorders). Furthermore, 90 % of patients in this study underwent psychological evaluation and were accurately assessed for ID, with 16 of the 36 patients with mild ID being newly diagnosed at the time of admission. Moreover, the inclusion of borderline ID in this group may have contributed to its high percentage. More than half of the patients admitted to psychiatric EMU had no comorbid mental and behavioral problems, and 40 % of these patients were referred from non-psychiatric practices. Therefore, the establishment of psychiatric EMUs may improve access to EMUs for patients both with and without psychiatric comorbidities.

Patients were admitted to the EMU to confirm the diagnosis, select an optimal medication, and localize the ictal focus in candidates for epilepsy surgery as primary indications. Recording habitual seizures during LTVEM is required to fulfill the goal of admission, regardless of the purpose. The seizure recording rate in this study was 69 %. The possibility of seizure recording was higher when the seizure frequency was greater than weekly. The comorbidity of ID was associated with a higher likelihood of seizure recording, particularly in patients with more severe epilepsy. Previous studies have reported 46 %–91 % event capture rates during LTVEM [9], [12], [13], [14]. These differences can be explained by different epilepsy subtypes or admission purposes, different provocative methods, or different durations of LTVEM. Nonetheless, the results are generally assumed to be similar to those of previous studies.

The change in diagnosis before and after LTVEM had a significant clinical impact and was an important indicator of the effectiveness of the EMU. Previous studies have reported 24 %–61 % changed diagnoses after LTVEM [15], [16], [17], [18]. These variations can be influenced by differences in the purpose of LTVEM or different meanings of “change in diagnosis, such as ES versus non-ES or focal ES versus general ES. Alsaddi et al. reported a change in diagnosis (without including a change in the ES type) in 29 of 121 patients (24 %) [15], with a diagnosis change rate similar to that in our study (28 %). In the diagnostic category, a decrease in epilepsy cases and an increase in non-epilepsy cases after LTVEM was consistent with previous reports [16], [18].

The adjustment of ASM therapy based on EMU findings is also an important factor in assessing the effectiveness of EMU. However, the prognosis of ES treated with ASMs after EMU evaluation has not been well documented. Dobesberger et al. reported that 28 % of the patients were seizure-free or exhibited > 50 % improvement. However, the percentage of patients treated with ASMs alone was not known because their patients included surgical cases [19]. Previous observational studies involving cohorts with medically refractory seizures receiving ASMs in academic epilepsy centers have reported 12 %–17 % of seizure-free cases with the addition of untried ASMs [20], [21]. Although whether EMU was also performed in these studies remains unclear, the superiority of the seizure-free rate in our study (24 %) may reflect the effectiveness of the EMU in pharmacotherapy. The recording and precise diagnosis of different seizure types through EMU evaluation allows clinicians to select optimal medications [22]. Moreover, viewing the recorded seizure videos with patients and their families while explaining clinical results on the day of discharge may improve treatment adherence.

Moreover, EMU is an essential component of pre-surgical evaluation to determine whether individuals with drug-resistant epilepsy are candidates for epilepsy surgery [22].

In this study, although 11 patients (8 %), including 4 admitted for pre-surgical evaluation, eventually underwent surgical procedures, no further LTVEM was necessary.

Unlike usual hospitalization for seizure cessation or reduction, the purpose of EMU admission is to record the seizures. Therefore, EMU admission can increase the risk of AEs, making it even more important to monitor the safety and utility of EMUs. Previous studies have reported AEs, such as falls, seizure-related injury, SE, medication-related AEs, seizure clusters, post-ictal psychosis, and cardiorespiratory complications, in patients admitted to EMUs [9], [23]. A previous systematic review on the safety of EMU admission reported a 7 % pooled estimate of AEs, with significant heterogeneity [23]. In this study, AEs in the EMU occurred in 9.7 % of cases, with falls being the most frequent, followed by seizure clusters and panic attacks. This higher rate can be explained by three factors. First, our study may have enrolled a higher percentage of patients with psychiatric comorbidities (47 %). A previous study reported a 16-fold increased risk of developing psychiatric AEs in an EMU in patients with psychiatric comorbidities [19]. In this study, 3 of the 13 AEs were psychiatric AEs (panic attacks). Two of the three patients had a history or comorbidity of anxiety, but one had no history or comorbidity of psychiatric symptoms. In contrast to previous studies that reported post-ictal psychosis as the most common psychiatric AE [19], [24], no cases of post-ictal psychosis were observed in our study. This is presumably because the psychiatrists and experienced psychiatric nurses carefully observed the patients with pre-existing psychiatric symptoms or those at risk for post-ictal psychosis to manage them during a cluster of seizures. In contrast, many panic attack cases were observed, likely due to comorbid anxiety disorders, pressures to experience seizures, and the inability to leave the room. Second, the longer hospital stay in this study may have contributed to the high rate of AEs. Many patients underwent various tests other than LTVEM, and half of the patients who fell did not undergo LTVEM. Measures should be taken to avoid falls during transportation after LTVEM, for example, by checking for a history of falls, having a staff member accompany the patient during ambulation, or using a wheelchair. Third, the lack of personnel to continuously monitor the patient’s condition for 24 h during LTVEM may also be a factor. The National Association of Epilepsy Centers in the United States recommends continuous, real-time supervision of ongoing LTVEM in EMUs [25]. Many United States epilepsy centers have trained technologists performing 24-h continuous monitoring [9]. The three falls during LTVEM in this study could have been better managed with real-time monitoring.

The three upsetting incidents following prolonged PNES with motor symptoms could have been influenced by the shared room environment between the patient and family member. A previous study showed that more than half of PNES cases, but not ES, could be alleviated or intensified by the presence of others [26]. Although PNES can be sustained by interactions between patients and companions, having companions present during LTVEM was considered preferable in our setting. Therefore, a careful review of the symptoms in patients with suspected PNES, discussion of the impact of the presence or absence of family members, and the preparation of a personalized protocol for PNES is necessary.

### 4.1 Study limitations

This study had some limitations. First, family members were encouraged to attend the EMU for safety purposes. In Japan, the reimbursement claims for EMU admission are lower than those in Europe and the United States, making it difficult to hire dedicated EMU medical staff [5]. Therefore, not only psychiatric but also general wards often request a family member to accompany the patient during EMU stays. Furthermore, the Mental Health and Welfare Law imposes very strict restrictions on physical restraints in psychiatric wards. Therefore, patients at high risk of falls due to seizures, but without a companion, were not considered suitable for psychiatric EMU admission, which may have potentially introduced patient selection bias. Although the presence of a family member can influence PNES frequency and duration, their absence may prevent adequate ASM dose reduction, resulting in a failure to document seizures. Second, the number of cases was small and the observation period short in this study. More than half of the PNES cases in this study were not confirmed via video-EEG but were diagnosed comprehensively based on the results of psychological and other tests. In some cases, the diagnosis may change over a long follow-up period. A previous study in which ASMs were tapered off in patients with PNES who were considered to have no history or complications of epilepsy reported that ES occurred in 3 of 73 patients during a 1-year follow-up period [27]. Therefore, the observation period of 6 months may be insufficient for patients with annual seizures. Third, the impact of EMU on ES/PNES control was not fully investigated in terms of the factors other than LTVEM. For example, MRI and other imaging tests may also influence diagnoses and treatment plans. Furthermore, the psychosocial life assessment conducted by the psychiatrists, psychologists, and other multidisciplinary professionals may also have impacted patients. Moreover, the presentation of the seizure videos and the explanation of the psychological evaluation at the time of discharge may have facilitated acceptance of the diagnosis, particularly in patients with PNES. Many patients with PNES could start psychiatric treatment in our hospital after EMU. Future investigations into the therapeutic effects of these assessments and the influence of interventions other than LTVEM are warranted.

## 5 Conclusions

Admission to an EMU can facilitate an accurate diagnosis and improvement in the management of epilepsy, both in psychiatric and general settings. In terms of safety, enhanced fall prevention measures during and after LTVEM may lead to safer EMU stays in psychiatric wards. More psychiatric wards with EMUs are expected to contribute to improved access to specialized epilepsy care for patients with comorbid psychiatric disorders and provide valuable experience and knowledge on epilepsy to young psychiatrists.

## CRediT authorship contribution statement

**Go Taniguchi:** Writing – original draft, Project administration, Investigation, Data curation, Conceptualization. **Mao Fujioka:** Writing – review & editing, Visualization, Formal analysis, Data curation. **Yumiko Okamura:** Writing – review & editing, Methodology, Data curation. **Minako Miyagi:** Writing – review & editing. **Kenichi Yano:** Writing – review & editing. **Shinsuke Kondo:** Writing – review & editing. **Kiyoto Kasai:** Supervision.

## Funding

This research did not receive any specific grant from funding agencies in the public, commercial, or not-for-profit sectors.

## Declaration of competing interest

The authors declare that they have no known competing financial interests or personal relationships that could have appeared to influence the work reported in this paper.

## Data Availability

Raw data were generated at the Department of Neuropsychiatry, The University of Tokyo Hospital. Derived data supporting the findings of this study are available from the corresponding author (G.T) upon request.
