# Supplementary material for: Utility and safety of epilepsy monitoring unit in an inpatient psychiatric setting in Japan
Source: Epilepsy Behav Rep. 2025 Jan 29;29:100744. doi: 10.1016/j.ebr.2025.100744 (PMC11869953; doi:10.1016/j.ebr.2025.100744)
Supplement: Supplementary Data 1 [file mmc1.docx]

**Supplement file 1.** **Comparison of the patients with adverse events and without adverse events in the epilepsy monitoring unit in psychiatry.**

|  | AE group (N=13) | Non-AE group (N=121) | P-value |
| --- | --- | --- | --- |
| Sex, female | 10 (77%) | 69 (57%) | 0.166 |
| Age on admission (years) | 34.2 | 35.5 | 0.755 |
| psychiatric comorbidities | 6 (46%) | 56 (46%) | 0.993 |
| intellectual disabilities | 10 (77%) | 70 (58%) | 0.183 |
| physical disabilities | 1 (8%) | 8 (7%) | 1.000 |
| daily seizure | 2 (15%) | 12 (10%) | 0.627 |
| weekly seizure | 6 (46%) | 43 (36%) | 0.547 |
| monthly seizure | 5 (38%) | 54 (45%) | 0.774 |
| yearly seizure | 0 (0%) | 10 (8%) | 0.597 |
| LTVEM (days) | 4.6 | 4.7 | 0.764 |
| Total admission (days) | 11.2 | 11.1 | 0.868 |
| companion | 12 (92%) | 97 (80%) | 0.461 |
| Withdrawal of ASM | 11 (85%) | 104 (86%) | 1.000 |

For categorial variables, we conducted Chi square or Fisher’s exact test according to the expected frequency in the cell. For univariate analysis, we used a parametric test (Student’s t-test) when the variables had a normal distribution and a nonparametric test (Mann-Whitney test) for a non-normal distribution. Age on admission and LTVEM duration are shown as means

Abbreviations: AE, adverse events; LTVEM, long-term video electroencephalographic monitoring; PNES, psychogenic non epileptic seizure. Significance set at 0.05.
